# Supplementary material for: RNase H2, mutated in Aicardi‐Goutières syndrome, resolves co-transcriptional R-loops to prevent DNA breaks and inflammation
Source: Nat Commun. 2022 May 26;13:2961. doi: 10.1038/s41467-022-30604-0 (PMC9135716; doi:10.1038/s41467-022-30604-0)
Supplement: Supplementary file 1 — Supplementary Information [file 41467_2022_30604_MOESM1_ESM.pdf]

RNase H2, mutated in Aicardi-Goutières syndrome, resolves co-transcriptional R-loops to prevent DNA breaks and inflammation.

Agnese Cristini[1], Michael Tellier[1], Flavia Constantinescu[1], Clelia Accalai[1], Laura Oana Albulescu[1], Robin Heiringhoff[1], Nicolas Bery[2], Olivier Sordet[3], Shona Murphy[1], Natalia Gromak[1,4]

[1]Sir William Dunn School of Pathology, University of Oxford, South Parks Road, Oxford, OX1 3RE, UK

[2]Weatherall Institute of Molecular Medicine, MRC Molecular Haematology Unit, University of Oxford, John Radcliffe Hospital, Oxford, OX3 9DS UK

[3]Cancer Research Center of Toulouse, INSERM, Université de Toulouse, Université Toulouse III Paul Sabatier, CNRS, 31037 Toulouse, France

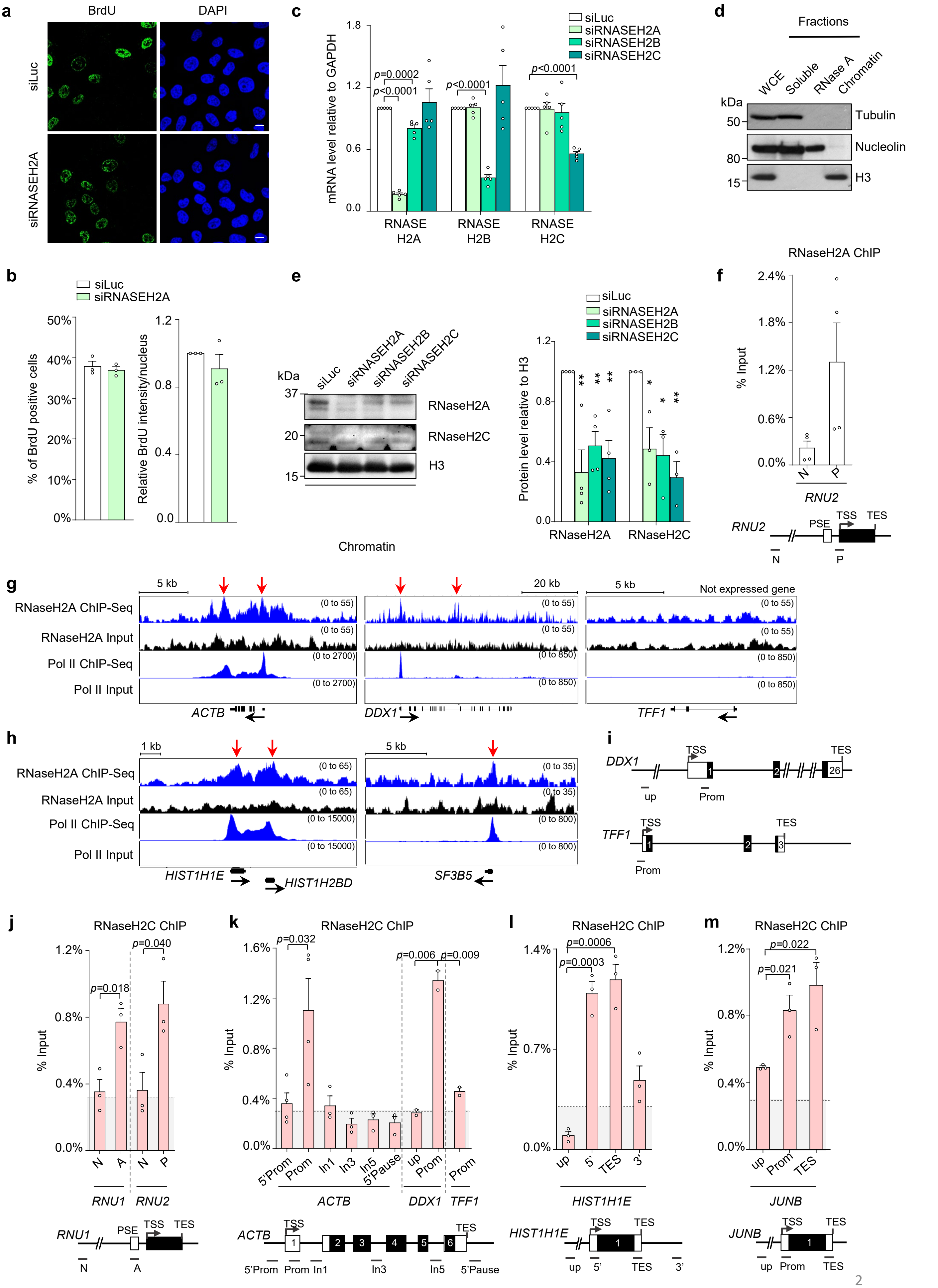
a
BrdU
DAPI
siLuc
siRNASEH2A
b
siLuc
siRNASEH2A
% of BrdU positive cells
Relative BrdU intensity/nucleus
c
siLuc
siRNASEH2A
siRNASEH2B
siRNASEH2C
mRNA level relative to GAPDH
p=0.0002
p<0.0001
p<0.0001
p<0.0001
RNASE H2A
RNASE H2B
RNASE H2C
d
Fractions
WCE
Soluble
RNase A
Chromatin
kDa
Tubulin
Nucleolin
H3
e
kDa
siLuc
siRNASEH2A
siRNASEH2B
siRNASEH2C
RNaseH2A
RNaseH2C
H3
Chromatin
Protein level relative to H3
RNaseH2A
RNaseH2C
f
RNaseH2A ChIP
% Input
N
P
RNU2
PSE
TSS
TES
g
5 kb
20 kb
5 kb
Not expressed gene
RNaseH2A ChIP-Seq
RNaseH2A Input
Pol II ChIP-Seq
Pol II Input
ACTB
DDX1
TFF1
h
1 kb
5 kb
HIST1H1E
HIST1H2BD
SF3B5
i
DDX1
TSS
TES
up
Prom
TFF1
j
RNaseH2C ChIP
% Input
p=0.018
p=0.040
RNU1
RNU2
A
k
RNaseH2C ChIP
p=0.032
p=0.006
p=0.009
5'Prom
Prom
In1
In3
In5
5'Pause
ACTB
l
RNaseH2C ChIP
p=0.0006
p=0.0003
up
5'
TES
3'
HIST1H1E
m
RNaseH2C ChIP
p=0.022
p=0.021
JUNB

Supplementary Figure 1. RNase H2A and RNase H2C binding to chromatin and transcribed genes.
a-b HeLa cells transfected with siLuc (white bars) or siRNASEH2A (green bars) were incubated with BrdU (10 µM, 1h) before staining. Representative pictures; BrdU (green), DAPI (blue); scale bars: 10 µm.
b Quantification of BrdU positive cells (left panel) and BrdU intensity per nucleus (right panel). >80 nuclei were quantified per condition (means ± SEM; *n*=3 biologically independent experiments).
c HeLa cells were transfected with siLuc (white) or siRNASEH2A, RNASEH2B and RNASEH2C (shades of green). qRT-PCR of indicated mRNAs; values are relative to siLuc cells (means ± SEM; *n*=5 biologically independent experiments). *p*-values were calculated using two-tailed unpaired *t*-test.
d HeLa cells were subjected to fractionation and proteins from the whole cell extracts (WCE), 'soluble', 'RNase A' and 'chromatin' fractions were analyzed by Western blot. Tubulin, nucleolin and H3 are corresponding controls for the soluble, RNase A and chromatin fractions. Representative blots from *n*=2 biologically independent experiments.
e Western blot of RNase H2A and H2C in the chromatin fraction of HeLa cells transfected with siLuc, siRNASEH2A, siRNASEH2B and siRNASEH2C (shades of green). H3 is a loading control (left panel). Quantification of protein levels (right panel). Values are normalized to H3 (means ± SEM); *n*=4 (RNase H2A) and *n*=3 (RNase H2C) biologically independent experiments. *p*-values from left to right: *p*=0.0043, *p*=0.0029, *p*=0.0020, *p*=0.0213, *p*=0.0172, *p*=0.0025 (two-tailed unpaired *t*-test).
f RNase H2A ChIP-qPCR on *RNU2* in HeLa cells. Values are expressed as percentage of input (means ± SEM; *n*=4 biologically independent experiments). All subsequent diagrams of the indicated genes are represented on the bottom panel and depict snRNA encoding region or protein-coding gene exons (black), UTRs (white), introns (lines), TSS, TES and qPCR amplicons (below the diagram). PSE is proximal sequence element.
g-h RNase H2A and Pol II ChIP-seq profiles on different gene categories in HeLa cells. **g** Intron-containing genes (*ACTB*, *DDX1*, *TFF1*: not expressed). **h** Histone (*HIST1H1E* and *HIST1H2BD*) and intronless (*SF3B5*) genes. Red arrows highlight RNase H2A enrichment over background and correlation with Pol II occupancy. For all profiles, numbers in brackets indicate the viewing range (FPKM).
i Diagrams of the indicated genes.
j-m RNase H2C ChIP-qPCR of different categories of genes in HeLa cells. **j** snRNA genes (*RNU1*, *RNU2*). **k** Intron-containing genes (*ACTB*, *DDX1* and *TFF1*). **l** Histone *HIST1H1E* gene. **m** Intronless *JUNB* gene. Values represent percentage of input (means ± SEM). *n*=3 biologically independent experiments (*n*=2 for *DDX1* and *TFF1*). *p*-values were calculated using two-tailed unpaired *t*-test.
Horizontal dotted line indicates background.

Source data are provided as a Source data file.

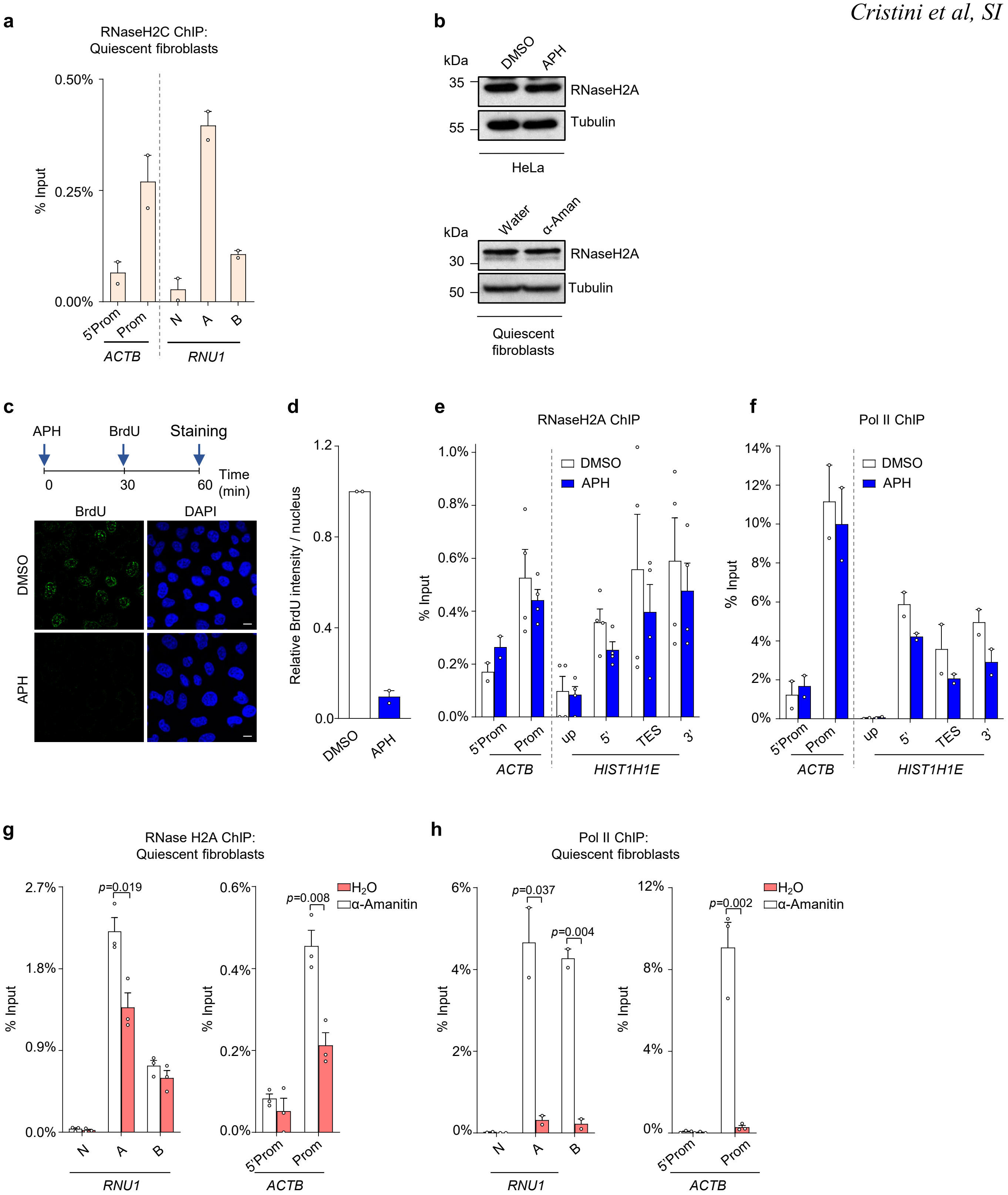
a
RNaseH2C ChIP:
Quiescent fibroblasts
% Input
0.50%
0.25%
0.00%
5'Prom
Prom
N
A
B
ACTB
RNU1
b
kDa
DMSO
APH
35
55
RNaseH2A
Tubulin
HeLa
kDa
Water
α-Aman
30
50
RNaseH2A
Tubulin
Quiescent
fibroblasts
c
APH
BrdU
Staining
0
30
60
Time
(min)
BrdU
DAPI
DMSO
APH
d
Relative BrdU intensity / nucleus
1.2
0.8
0.4
0.0
DMSO
APH
e
RNaseH2A ChIP
% Input
1.0%
0.8%
0.6%
0.4%
0.2%
0.0%
DMSO
APH
5'Prom
Prom
up
5'
TES
3'
ACTB
HIST1H1E
f
Pol II ChIP
% Input
14%
12%
10%
8%
6%
4%
2%
0%
DMSO
APH
5'Prom
Prom
up
5'
TES
3'
ACTB
HIST1H1E
g
RNase H2A ChIP:
Quiescent fibroblasts
% Input
2.7%
1.8%
0.9%
0.0%
p=0.019
N
A
B
RNU1
% Input
0.6%
0.4%
0.2%
0.0%
p=0.008
H2O
α-Amanitin
5'Prom
Prom
ACTB
h
Pol II ChIP:
Quiescent fibroblasts
% Input
6%
4%
2%
0%
p=0.037
p=0.004
N
A
B
RNU1
% Input
12%
8%
4%
0%
p=0.002
H2O
α-Amanitin
5'Prom
Prom
ACTB

Supplementary Figure 2. RNase H2 binding is replication-independent.
a RNase H2C ChIP-qPCR of *ACTB* and *RNU1* genes in quiescent fibroblasts. Values are expressed as percentage of input (means ± SEM; *n*=2 independent biological experiments).
b Western blot of RNase H2A in HeLa cells treated with aphidicolin (APH; 1 µM, 1 h) (top panel) and in quiescent fibroblasts treated with α-amanitin (2.5 µg/ml, 24 h) (bottom panel). The corresponding vehicles (DMSO and water) are indicated. Tubulin is used as loading control. Representative blots from *n*=1 biologically independent experiment.
c-d HeLa cells were treated with aphidicolin (APH, 1 µM, 1 h) 30 min before the addition of BrdU (100 µM) to the medium for 30 min. Cell treatment protocol (top panel) and representative pictures (bottom panel); BrdU (green), DAPI (blue); scale bars: 10 µm. **d** Quantification of BrdU intensity per nucleus in DMSO- (white bars) and APH- (blue bars) treated cells (means ± SEM; *n*=2 biologically independent experiments); >70 nuclei were quantified per condition.
e RNase H2A and **f** Pol II ChIP-qPCR of *ACTB* and *HIST1H1E* genes in HeLa cells treated with DMSO (white bars) or aphidicolin (APH, 1 µM, 1 h; blue bars). Values are expressed as percentage of input (means ± SEM). *n*=4 (*n*=2 for *ACTB* 5'Prom) in **e** and *n*=2 biologically independent experiments in **f**.
g RNase H2A and **h** Pol II ChIP-qPCR of *RNU1* and *ACTB* genes in quiescent fibroblasts treated with H2O (white bars) or α-amanitin (2.5 µg/ml, 24 h, pink bars). Values are expressed as percentage of input (means ± SEM). *n*=3 (*n*=2 for *RNU1* in **h**) biologically independent experiments. *p*-values were calculated using two-tailed unpaired *t*-test.

Source data are provided as a Source data file.

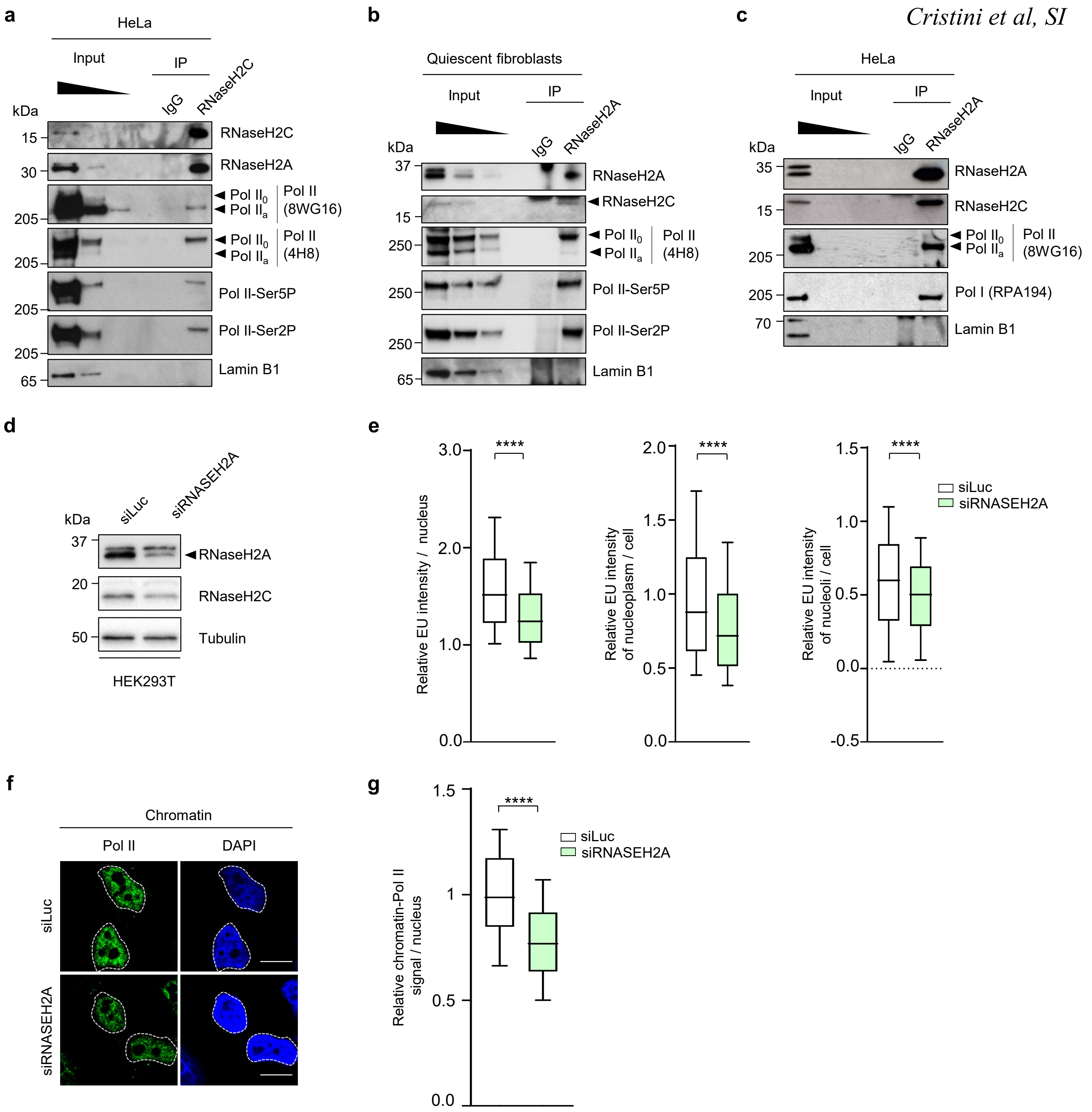
a
HeLa
Input
IP
IgG
RNaseH2C
kDa
15
30
205
205
205
205
65
RNaseH2C
RNaseH2A
Pol II0
Pol IIa
Pol II (8WG16)
Pol II (4H8)
Pol II-Ser5P
Pol II-Ser2P
Lamin B1
b
Quiescent fibroblasts
Input
IP
IgG
RNaseH2A
kDa
37
15
250
250
250
65
RNaseH2A
RNaseH2C
Pol II0
Pol IIa
Pol II (4H8)
Pol II-Ser5P
Pol II-Ser2P
Lamin B1
c
HeLa
Input
IP
IgG
RNaseH2A
kDa
35
15
205
205
70
RNaseH2A
RNaseH2C
Pol II0
Pol IIa
Pol II (8WG16)
Pol I (RPA194)
Lamin B1
d
kDa
siLuc
siRNASEH2A
37
20
50
RNaseH2A
RNaseH2C
Tubulin
HEK293T
e
Relative EU intensity / nucleus
Relative EU intensity of nucleoplasm / cell
Relative EU intensity of nucleoli / cell
****
3.0
2.0
1.0
0.0
1.5
0.5
-0.5
siLuc
siRNASEH2A
f
Chromatin
Pol II
DAPI
siLuc
siRNASEH2A
g
Relative chromatin-Pol II signal / nucleus
1.5
1
0.5
0.0
****
siLuc
siRNASEH2A

Supplementary Figure 3. RNase H2 is in complex with Pol II and RNase H2A depletion affects global Pol II binding to chromatin.
a Western blot of endogenous RNase H2C IP from HeLa WCE. **b** RNase H2A IP from quiescent fibroblast WCE. **c** RNase H2A IP from HeLa WCE. Arrows indicate hypo- (IIa) and hyper- (IIo) phosphorylated forms of Pol II. IgG and Lamin B1 are negative controls. Representative blots from n=2 (**a**, **b**) or n=1 (**c**) biologically independent experiments.
d Western blot of RNase H2A and H2C in the whole cell extract (WCE) in HEK293T cells transfected with siLuc or siRNASEH2A. Tubulin is a loading control. Representative blots from n=2 biologically independent experiments.
e Quantification of EU intensity in HEK293T cells transfected with siLuc (white bars) or siRNASEH2A (green bars) per nucleus (left panel), per nucleoplasm (nucleus without nucleolus signal per cell; middle panel) or per nucleoli (signal per cell; right panel). EU intensity per cell is normalized to the average nucleoplasmic intensity in siLuc condition. Boxplot settings are: box: 25-75 percentile range; whiskers: 10-90 percentile range; horizontal bars: median; outliers not displayed. Representative data from n=2 biologically independent experiments. > 50,000 nuclei were quantified per condition. ****p<0.0001 (two-tailed unpaired t-test).
f-g IF analysis of chromatin-bound Pol II in HeLa cells following RNase H2A depletion and removal of soluble nuclear proteins before the staining. **f** Representative pictures; EU (green), DAPI (blue); scale bars: 10 µm. **g** Quantification of Pol II intensity per nucleus; siLuc: white bar, siRNASEH2A: green bar. Boxplot settings are: box: 25-75 percentile range; whiskers: 10-90 percentile range; horizontal bars: median; outliers not displayed. Representative data from n=2 biologically independent experiments. >100 nuclei were quantified per condition; ****p<0.0001 (two-tailed unpaired t-test).

Source data are provided as a Source data file.

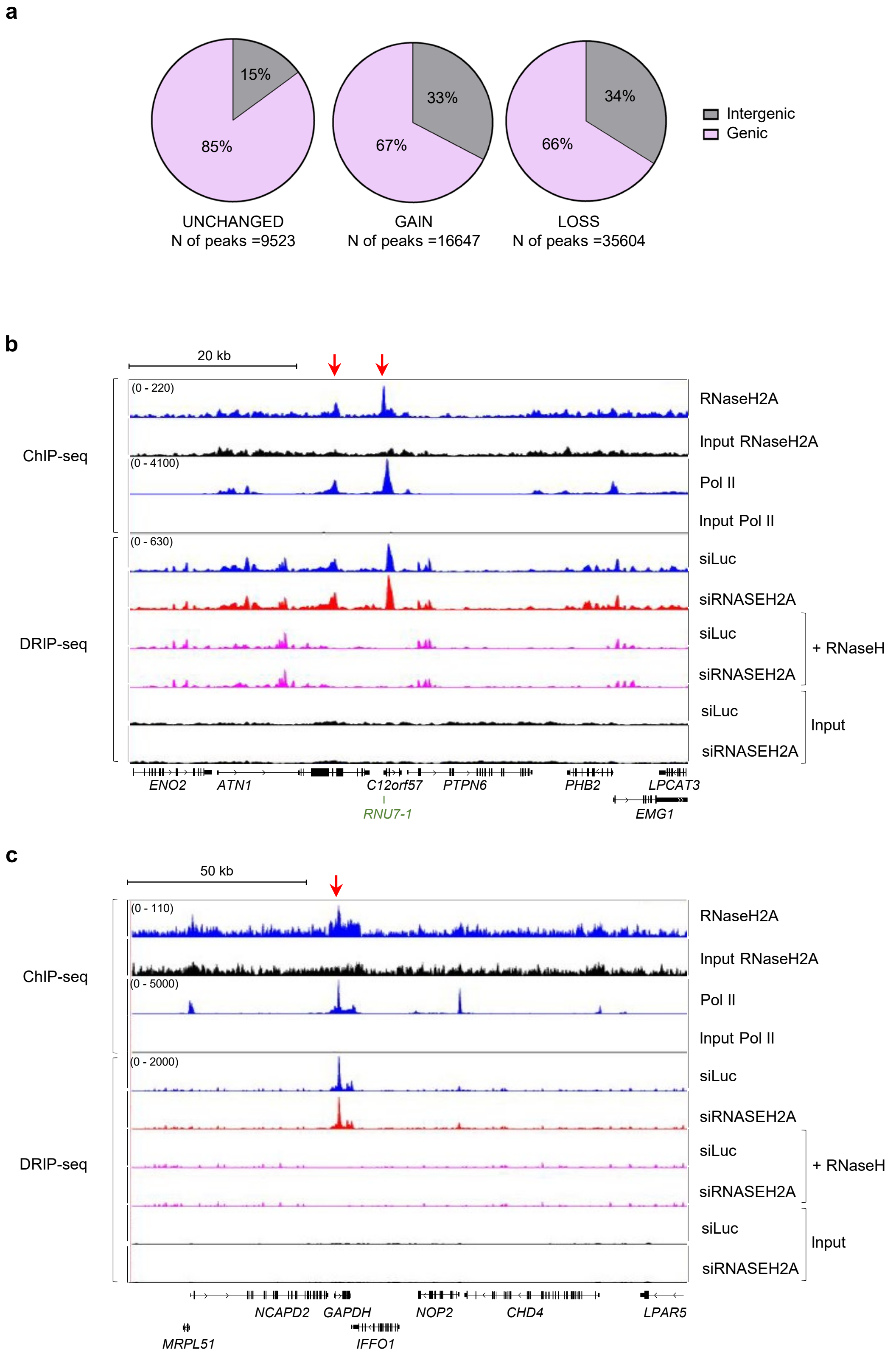

Supplementary Figure 4. Genome-wide R-loop changes in RNase H2 depleted cells.
a DRIP-seq peak distribution across genic and intergenic compartments in control and RNase H2A-depleted HeLa cells. The number below each pie graph represents the total number of DRIP peaks in the unchanged (left panel), increased/gained (middle panel) and decreased/lost (right panel) categories in RNase H2A-depleted HeLa cells.
b-c RNase H2A ChIP-seq, Pol II ChIP-seq and DRIP-seq profiles of multiple genes in untreated HeLa (ChIP-seq) or HeLa cells transfected as in **a**.

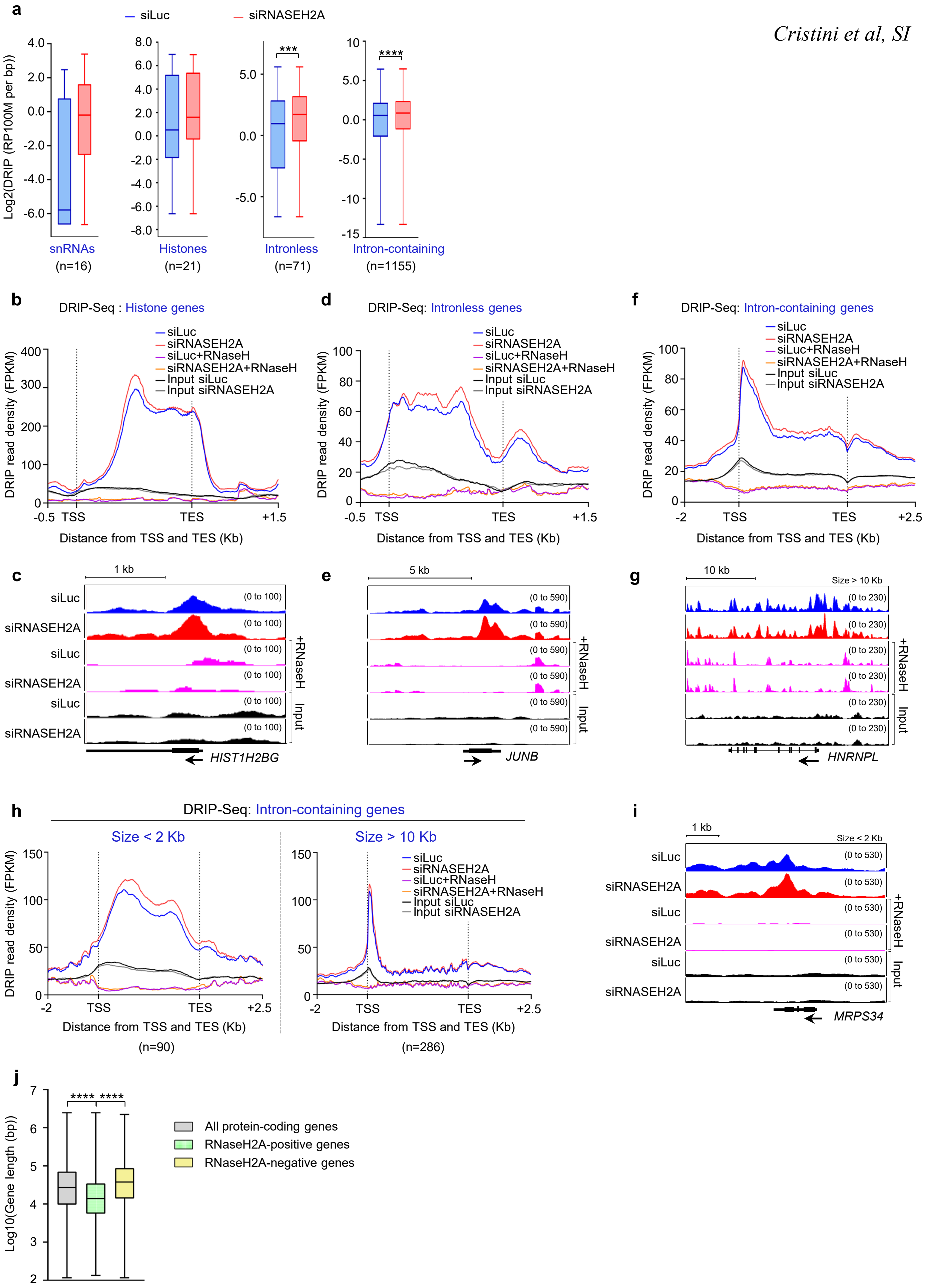
a
siLuc
siRNASEH2A
Log2(DRIP (RP100M per bp))
snRNAs
(n=16)
Histones
(n=21)
Intronless
(n=71)
Intron-containing
(n=1155)
***
****
b
DRIP-Seq : Histone genes
d
DRIP-Seq: Intronless genes
f
DRIP-Seq: Intron-containing genes
siLuc
siRNASEH2A
siLuc+RNaseH
siRNASEH2A+RNaseH
Input siLuc
Input siRNASEH2A
DRIP read density (FPKM)
Distance from TSS and TES (Kb)
TSS
TES
c
1 kb
(0 to 100)
HIST1H2BG
e
5 kb
(0 to 590)
JUNB
g
10 kb
Size > 10 Kb
(0 to 230)
HNRNPL
+RNaseH
Input
h
DRIP-Seq: Intron-containing genes
Size < 2 Kb
Size > 10 Kb
(n=90)
(n=286)
i
1 kb
Size < 2 Kb
(0 to 530)
MRPS34
j
Log10(Gene length (bp))
**** ****
All protein-coding genes
RNaseH2A-positive genes
RNaseH2A-negative genes

Supplementary Figure 5. R-loops accumulate in short genes upon RNase H2 depletion.

a Boxplots of DRIP-seq signals across snRNAs (n=16), histones (n=21), intronless (n=71) and intron-containing (n=1155) genes in control (blue) and RNase H2A-depleted (red) HeLa cells. To select genes strongly enriched for R-loops, only genes with average signal over two biologically independent experiments enriched versus input and RNase H-treated control are included. Boxplot settings are: box: 25-75 percentile range; whiskers: minimal to maximal values; horizontal bars: median; outliers not displayed. ***p=0.0002, ****p<0.0001 (Two-tailed Wilcoxon matched-pairs signed rank-test)

b, d, f Meta-analysis of DRIP-seq on different categories of genes in HeLa cells following RNase H2A depletion with the window size of the presented plots adjusted to the gene size. **b** Histone genes. **d** Intronless genes. **f** Intron-containing genes. All subsequent meta-analysis and profiles employing siRNASEH2A versus siLuc are shown in red and blue, respectively, and represent the average signal over two replicates (RNase H-treated control corresponds to one replicate).

c, e, g DRIP-seq profiles of *HIST1H2BG*, *JUNB* and *HNRNPL* genes in HeLa cells treated as in **b, d, f**.

h Meta-analysis of DRIP-seq on short (<2kb; left panel) and long (>10kb; right panel) intron-containing genes in HeLa cells following RNase H2A depletion with the window size of the presented plots adjusted to the gene size.

i DRIP-seq profiles of short intron-containing *MRPS34* gene in HeLa cells treated as in **b, d, f**

j Boxplots depicting distribution of lengths for all protein-coding (grey; n=19,909), ChIP-seq RNase H2A-positive (green; n=6,646) or ChIP-seq RNase H2A-negative (yellow; n=13,262) genes. Boxplot settings are: box: 25-75 percentile range; whiskers: minimal to maximal values; horizontal bars: median; outliers not displayed. ****p<0.0001 (Kruskal-Wallis test).

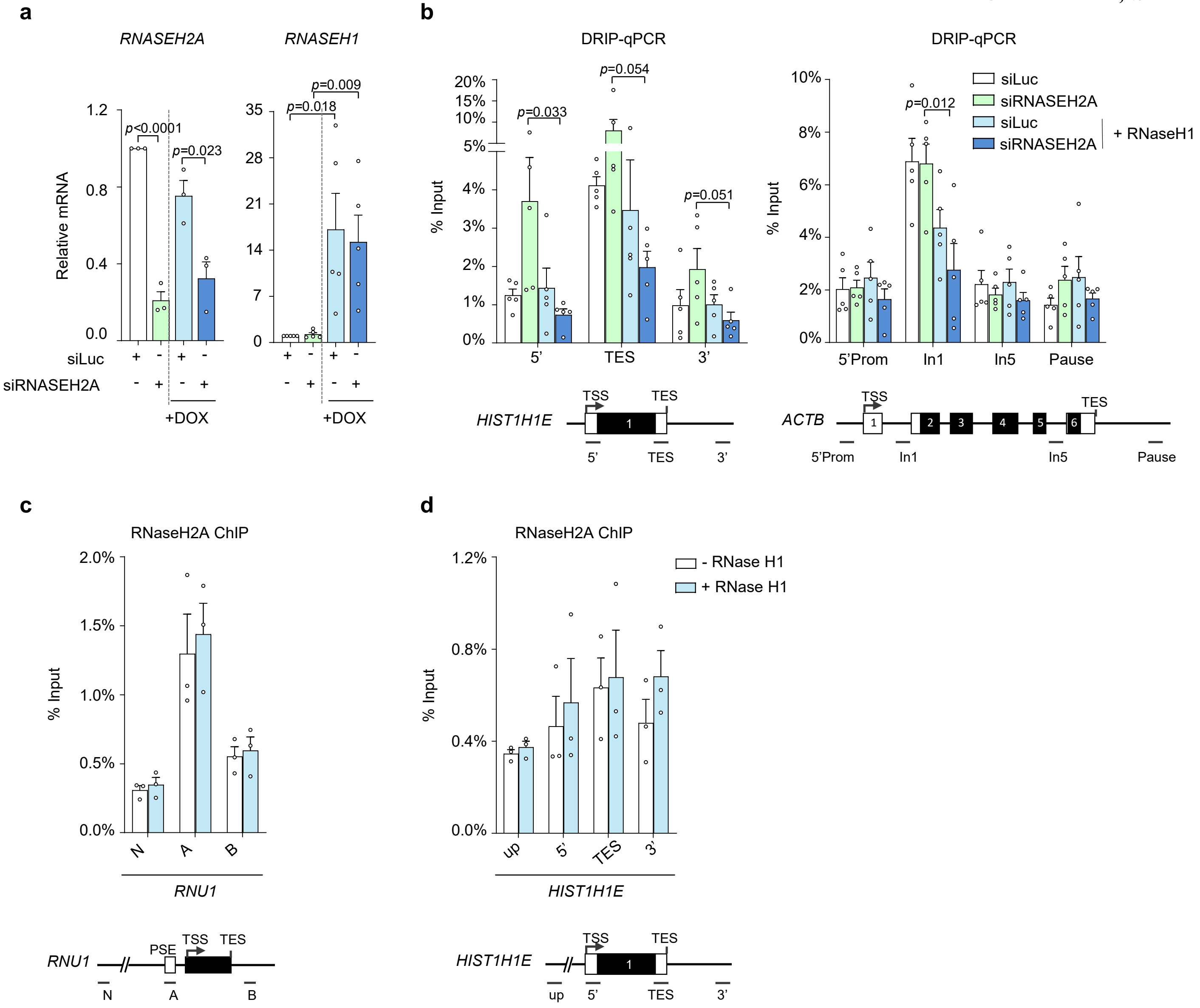

Supplementary Figure 6. RNase H1 overexpression reduces R-loops in RNase H2A-depleted cells.

a HEK293T RNase H1-FLAG-IRES-mCherry cells were depleted for RNase H2A (green bars) and induced with doxycycline (+DOX; shades of blue) as in Figure 4g. qRT-PCR of *RNASEH2A* (left panel) and *RNASEH1* (right panel) mRNAs; values are relative to siLuc cells; (means ± SEM). *n*=3 (for *RNASEH2A)* and *n*=5 (for *RNASEH1)* biologically independent experiments. *p*-values were calculated using two-tailed unpaired *t*-test.

b DRIP-qPCR for the indicated genes in HEK293T RNase H1-FLAG-IRES-mCherry cells following RNase H2A depletion (green bars) and RNase H1 doxycycline induction (shades of blue) as in Figure 4g. Values are expressed as percentage of input (means ± SEM). *n*=5 biologically independent experiments. *p*-values were calculated using two-tailed unpaired *t*-test. Gene diagrams are shown on the bottom panel.

c-d RNase H2A ChIP-qPCR in HEK293T RNase H1-FLAG-IRES-mCherry cells following RNase H1 doxycycline induction (blue bars) as in Figure 4g. Values are expressed as percentage of input (means ± SEM). *n*=3 biologically independent experiments. Gene diagrams are shown on the bottom panel.

Source data are provided as a Source data file.

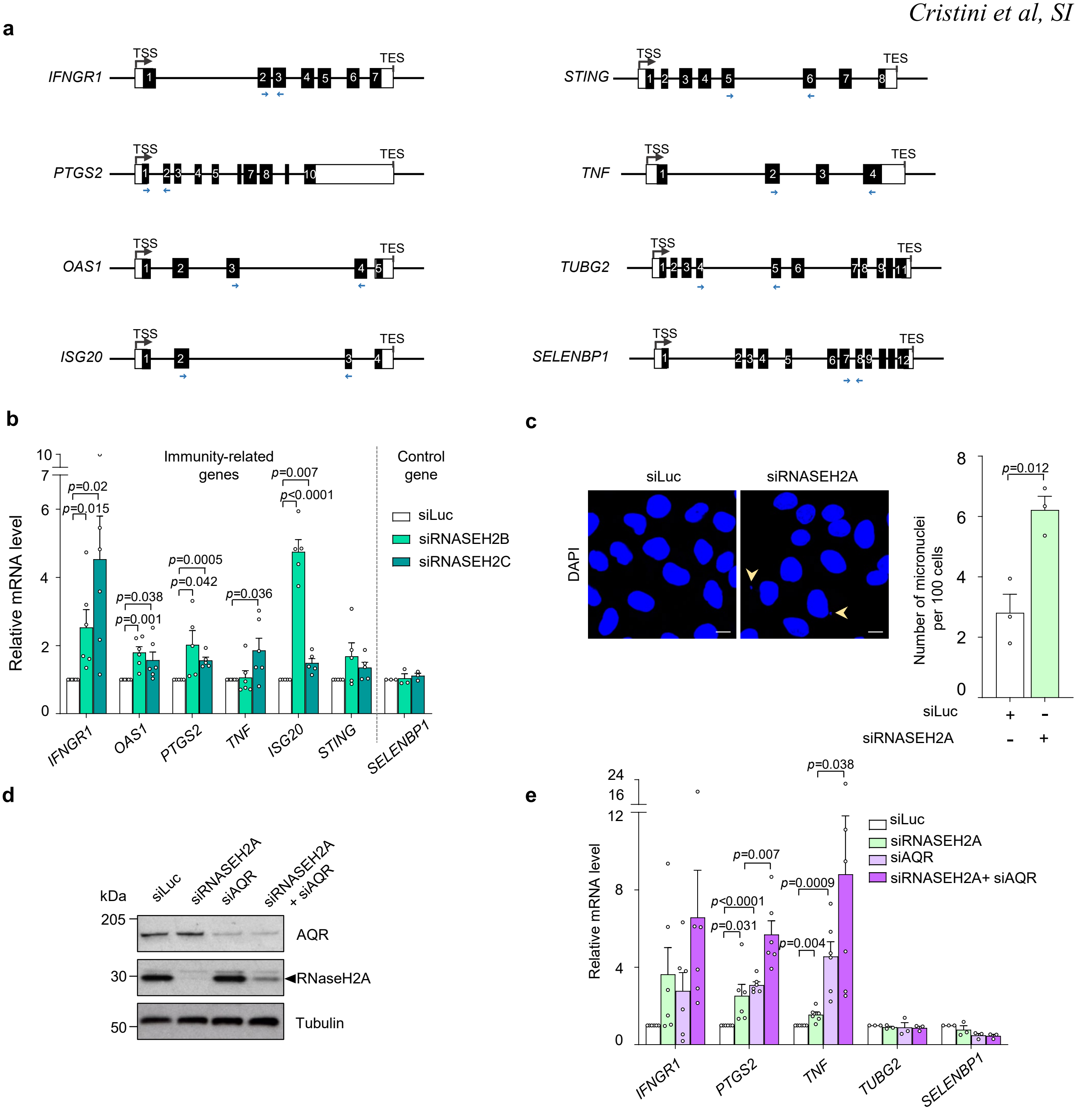

Supplementary Figure 7. Induction of immune-related genes in RNase H2-depleted cells.

a Diagrams of the indicated genes. The location of primer pairs for mRNA amplification is indicated in blue.

b qRT-PCR analysis of indicated mRNAs in HeLa cells following RNase H2B and RNase H2C depletion. Some replicates have been performed in parallel to siRNASEH2A in Fig. 5b. Values are relative to siLuc cells (means ± SEM). *n*=5 (*PTGS2, ISG20, STING*) or *n*=6 (*IFNGR1*, *OAS1*, *TNF*) biologically independent experiments. *n*=3 biologically independent experiments for the control *SELENPB1*. *p*-values were calculated using two-tailed unpaired *t*-test.

c IF analysis of micronuclei in HeLa cells following RNase H2A depletion. Representative pictures; DAPI (blue); scale bars: 10 µm; yellow arrows indicate micronuclei (left panel). Number of micronuclei per 100 cells (means ± SEM; *n*=3 biologically independent experiments). >14,000 nuclei were quantified per condition; *p*-value was calculated using two-tailed unpaired *t*-test.

d-e HeLa cells were transfected with the indicated siRNAs (siRNASEH2A alone is in green and siAQR samples are in shade of pink). **d** Western blot was probed with the indicated antibodies. **e** qRT-PCR analysis of indicated mRNAs; some replicates have been performed in parallel to siXPG and siXPF transfections in Fig. 5f and Supplementary Fig. 8f and have the same siLuc and siRNASEH2A controls. Values are relative to siLuc (means ± SEM). *n*=6 biologically independent experiments for *IFNGR1*, *PTGS2*, *TNF*. *n*=3 biologically independent experiments for the controls *TUBG2*, *SELENPB1*. *p*-values were calculated using two-tailed unpaired *t*-test. Source data are provided as a Source data file.

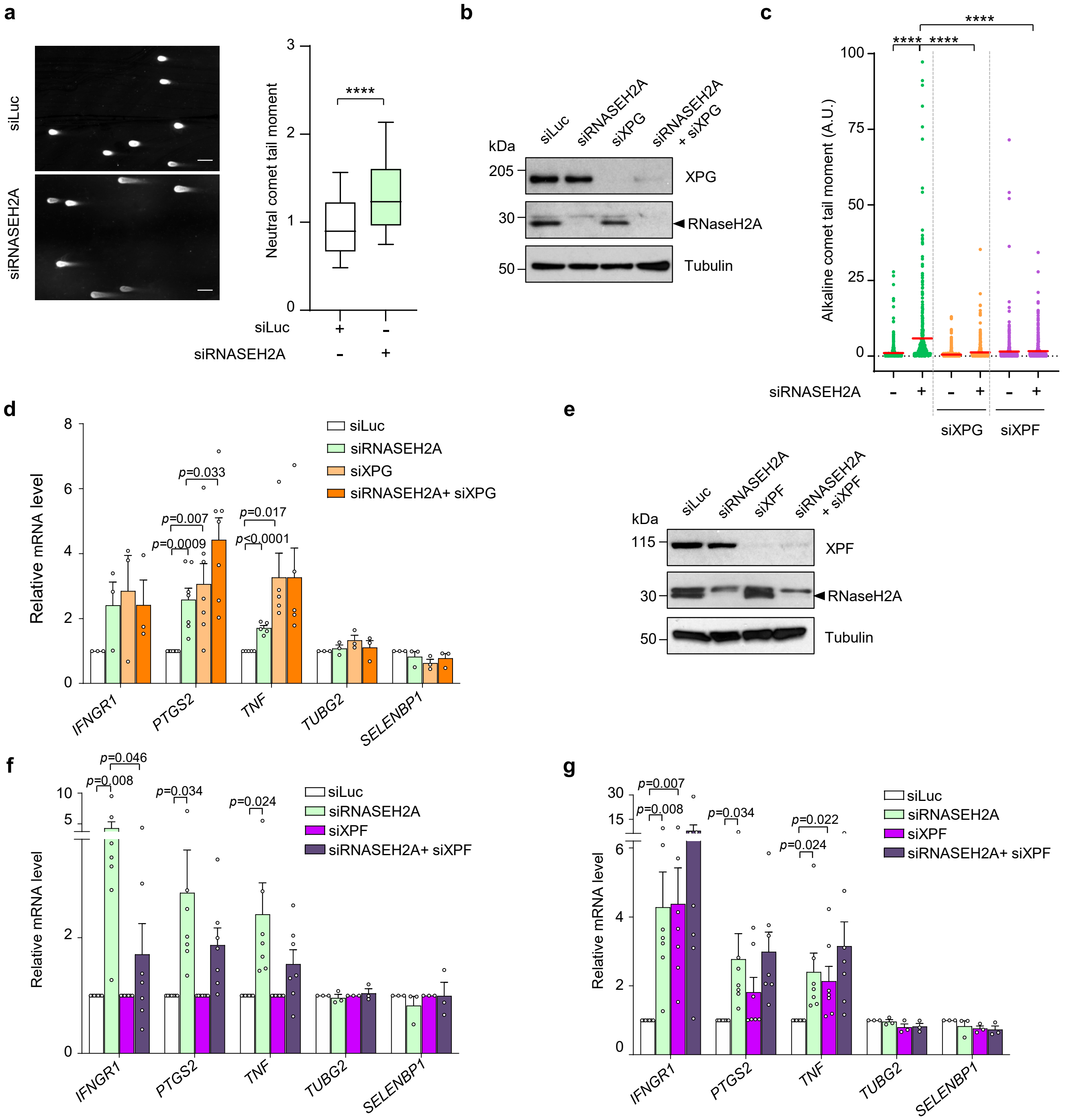

Supplementary Figure 8. Induction of DNA breaks in RNase H2-depleted cells.

a Neutral comet assays in HeLa cells following RNase H2A depletion. Representative images (left) and quantification of comet tail moment (right). Scale bars: 100 µm. Boxplot settings are: box: 25-75 percentile range; whiskers: 10-90 percentile range; horizontal bars: median; outliers not displayed. Representative data from n=2 biologically independent experiments. >650 nuclei were quantified per condition. **** p<0.0001 (two-tailed unpaired t-test).

b-g HeLa cells were transfected with the indicated siRNAs (siRNASEH2A alone is in green, siXPG are in shades of orange, siXPF in shades of purple). **b**, **e** Western blot was probed with the indicated antibodies. **c** Scatter dot plot representation of alkaline comet assay data from Fig. 5e. Horizontal bar indicates the mean. ****p<0.0001 (one-way ANOVA, Tukey's multiple comparison test). **d**, **f**, **g** qRT-PCR analysis of indicated mRNAs. Some replicates of siXPG and siXPF have been performed together or in parallel to siAQR transfections in Supplementary Fig.7e and have the same siLuc and siRNASEH2A controls. **d** Values from Fig. 5f are expressed as relative to siLuc (means ± SEM). n=3 (n=7 for *PTGS2* and n=5 for *TNF*) biologically independent experiments. **f** Values are expressed as relative to siLuc and siXPF cells (means ± SEM). n=7 biologically independent experiments. The controls *TUBG2*, *SELENPB1* are n=3. **g** Values from **f** are expressed as relative to siLuc (means ± SEM). p-values were calculated using two-tailed unpaired t-test. Source data are provided as a Source data file.

Supplementary Methods

Antibodies

The following antibodies were used for ChIP: Pol II (Novus, NBP2-32080; 1μg), RNase H2A (Proteintech, 16132-1-AP, Lot#00007351; ChIP 2.5 μg, ChIP-seq 4 μg) and RNase H2C (Proteintech, 16518-1-AP, Lot#00007806; ChIP 2.5 μg). Antibodies used for co-IP, western blotting and IF: AQR (Proteintech, 24342-1-AP; WB 1:500), BrdU clone B44 (BD Biosciences, #347580; IF 1:50), FLAG clone M2 (Sigma, CAT#F3165; WB 1:2000), H3 (Abcam, ab1791; WB 1:4000 or clone D1H2 Cell Signalling, #4499; WB 1:1000), IgG Isotype Control (Thermo Fisher, #02-6102; co-IP 2 μg), Lamin B1 (Abcam, ab16048; WB 1:2000), Nucleolin (Abcam, ab22758; IF and WB 1:1000), Pol I RPA194 clone C-1 (Santa cruz, sc-48385; WB 1:500), Pol II clone 8WG16 (Abcam, ab817; WB 1:1000), Pol II clone CTD4H8 (Santa Cruz, sc-47701; WB 1:1000), Pol II-S5P (Abcam, ab5131; WB 1:2000), Pol II-S2P (Abcam, ab5095; WB 1:2000), RNase H1 (Proteintech, 15606-1-AP; WB 1:1000), RNase H2A (Proteintech, 16132-1-AP; WB 1:1000; co-IP 2 μg), RNase H2C (Proteintech, 16518-1-AP; WB 1:500 and ChIP 2.5 μg), Tubulin (Abcam, ab4074; WB 1:5000), XPF (Abcam, ab76948; WB 1:500) and XPG (Proteintech, 11331-1-AP; WB 1:500). Fluorescent secondary antibody: donkey anti-mouse IgG (H+L) highly cross-adsorbed secondary antibody Alexa Fluor 488 (ThermoFisher, A-11001; IF 1:500), donkey anti-rabbit IgG (H+L) highly cross-adsorbed secondary antibody Alexa Fluor 594 (Life Technologies, A-21207; IF 1:500) or goat anti-rabbit IgG (H+L) highly cross-adsorbed secondary antibody Alexa Fluor 647 (Life Technologies, A-21245; IF 1:1000). S9.6 RNA/DNA hybrid antibody [1], produced in house, was used for DRIP-seq and DRIP-qPCR experiments (15 μl/IP).

Transcription units' annotation

Gencode V31 annotation, based on the hg38 version of the human genome, was used to extract the 18,872 protein-coding genes. All genes were taken from the most 5' TSS to the most 3' poly(A) site or transcription end site (TES). Intronless protein-coding genes (excluding histone genes), were defined as the protein-coding genes containing a single exon (921 genes). The histone genes were defined as the genes with the name starting with '*HIST'* (71 genes). snRNA genes were extracted from the Gencode V31 annotation by keeping only the genes, and not the pseudogenes, annotated as snRNA, corresponding to 35 genes. The eRNAs annotation based on the hg38 version of the human genome was taken from the FANTOM5 database. The list of intergenic eRNAs was obtained by keeping the eRNAs not overlapping with a transcription unit annotated in Gencode V31 (non-coding and protein-coding genes).

Chromatin RNA-seq data processing

Chromatin RNA-seq data were processed as described [2]. In brief, adapters were trimmed with Cutadapt version 1.13 [3] in paired-end mode with the following parameters: -q 15, 10 –minimum-length 10 -A

AGATCGGAAGAGCGTCGTGTAGGGAAAGAGTGT -a AGATCGGAAGAGCACACGTCTGAACTCCAGTCA. The remaining rRNA reads were removed by mapping the trimmed reads to the Human ribosomal DNA complete repeating unit (GenBank: U13369.1) with STAR version 2.6.1d [4] and the parameters --runThreadN 16 --readFilesCommand gunzip -c –k --outReadsUnmapped Fastx --limitBAMsortRAM 20000000000 --outSAMtype BAM SortedByCoordinate. The unmapped reads were mapped to the human hg38 reference genome with STAR and the parameters --runThreadN 16 --readFilesCommand gunzip -c –k --limitBAMsortRAM 20000000000 --outSAMtype BAM SortedByCoordinate. SAMtools version 1.3.1 [5] was used to retain only properly paired and mapped reads (-f 3). Strand-specific bam files were generated with SAMtools. FPKM-normalized bigwig files were created with deepTools version 2.5.0.1 [6] bamCoverage tool with the parameters –bs 10 –p max –-normalizeUsing RPKM.

ChIP-seq and DRIP-seq data processing

Adapters were trimmed with Cutadapt in paired-end mode with the same parameters as the Chromatin RNA-seq. Obtained sequences were mapped to the human hg38 reference genome with STAR and the parameters --runThreadN 16 --readFilesCommand gunzip -c –k --alignIntronMax 1 --limitBAMsortRAM 20000000000 --outSAMtype BAM SortedByCoordinate. Properly paired and mapped reads were filtered with SAMtools. PCR duplicates were removed with Picard MarkDuplicates tool. FPKM-normalized bigwig files were created with deepTools bamCoverage tool with the parameters –bs 10 –p max -e --normalizeUsing RPKM. DRIP-seq peaks were called from merged biological duplicates with MACS2 version 2.1.1.20160309 [7] and the parameters: callpeak -f BAMPE -g 2.9e9 -B -q 0.01 –call-summits. The total number of DRIP peaks for siLuc (56,997 peaks) and siRNASEH2A (50,955 peaks) were obtained by comparing each IP to its respective input. The 9,523 'unchanged', the 16,647 'increased/gained' and 35,604 'decreased/lost' DRIP-seq peaks were obtained after two analysis steps. First, the MACS2 bdgdiff tool was used to obtain the initial list of 'unchanged' (208,973 peaks), 'increased/gained' (3,119 peaks) and 'decreased/lost' (6,182 peaks) DRIP-seq peaks between siLuc and siRNASEH2A. This higher number of peaks compared to the total number of DRIP peaks for siLuc and siRNASEH2A is due to the difference in the peak calling algorithm used (MACS2 callpeak vs MACS2 bdgdiff) and the breakdown of the initial peaks into smaller peaks when one or more parts of an initial peak are enriched differently in siLuc or siRNASEH2A samples. Second, peak regions defined by MACS2 bdgdiff had to be superior in the IP samples compared to the IP+RNase H samples (see Reads quantification for the Method), as the IP+RNase H samples control for unequal non-R-loop background level in DRIP-seq. Following the inclusion of the IP+RNase H normalization, the remaining DRIP-seq peaks were classified again in 'unchanged' (9,523 peaks), 'increased/gained' (16,647 peaks) and 'decreased/lost' (35,604 peaks) based on the ratio of the reads quantification siRNASEH2A/siLuc (< 0.5: 'decreased/lost', >2: 'increased/gain', others: 'unchanged').

Metagene profiles

Metagene profiles were generated from FPKM-normalized bigwig files with Deeptools2 computeMatrix tool with a bin size of 10 bp and the plotting data obtained with plotProfile –outFileNameData tool. Graphs were then created with GraphPad Prism 8.3.1.

Reads quantification

Total read base count for chromatin RNA-seq data were computed with samtools bedcov tool using strand-specific bam files and normalized to 100 million paired-end reads and to the region's length. For ChIP-seq, total read base count were computed with samtools bedcov, normalized to 100 million paired-end reads, then the Input signal was subtracted from the IP signal and normalized to the region's length. Only the regions with a positive signal in at least one sample were kept. For the samples having a signal < 0 on the remaining regions, their values were put to the minimal value divided by two. For the overlap between RNase H2A ChIP-seq and the transcription units, including the intergenic eRNAs only, we considered as positive any transcription unit with an average RNase H2A signal superior to the Input signal across the TSS to the TES.

For DRIP-seq, total read base count were computed with samtools bedcov, normalized to 100 million paired-end reads, then the Input and the RNase H insensitive signal (DRIP-seq + RNAseH treatment) were subtracted from the IP signal and normalized to the region's length. Only the regions with a positive signal in at least one sample were kept (siLuc or siRNaseH2A). For the samples having a signal < 0 on the remaining regions, their values were put to the minimal value divided by two.

The quantification is thus defined: For chromatin RNA-seq: log2(([region] * normalization factor) / length region). For ChIP-seq: log2((([region]IP * IP normalization factor) - ([region]Input * Input normalization factor)) / length region). For DRIP-seq: log2(((([region]IP * IP normalization factor) - ([region]Input * Input normalization factor)) – ([region]IP+RNaseH * IP+RNaseH normalization factor) / length region). The quantification regions were defined as the TSS to the TES regions for the different group of genes. Box and whiskers were created with GraphPad Prism 8.3.1. with the following settings: boxes: 25-75 percentile range; whiskers: minimal to maximal values; horizontal bars: median.

Supplementary Table 1. List of primers used in this study.

**Name**	**Sequence 5'-3'**
**ChIP and DRIP**	
ACTB	
5'prom (F)	CCA CCT GGG TAC ACA CAG TCT
5'prom (R)	TGT CCT TGT CAC CCT TTC TTG
prom (F)	CCG AAA GTT GCC TTT TAT GGC
prom (R)	CAA AGG CGA GGC TCT GTG C
in1 (F)	CGG GGT CTT TGT CTG AGC
in1 (R)	CAG TTA GCG CCC AAA GGA C
in3(F)	TAA CAC TGG CTC GTG TGA CAA
in3(R)	AAG TGC AAA GAA CAC GGC TAA
in5(F)	GGA GCT GTC ACA TCC AGG GTC
in5(R)	TGC TGA TCC ACA TCT GCT GG
5'pause (F)	TTA CCC AGA GTG CAG GTG TG
5'pause (R)	CCC CAA TAA GCA GGA ACA GA
pause (F)	GGG ACT ATT TGG GGG TGT CT
pause (R)	TCC CAT AGG TGA AGG CAA AG
DDX1	
up (F)	AAT TTG GGG ATC CTG GCA GA
up (R)	TTG AGT GTT CGT CAG CCT CT
prom (F)	GGC CTT TCC TTC CAT CGG A
prom (R)	CAC AAA CGC ACC GGA GAA G
HIST1HE	
up (F)	AGT CCA TAA GCA GCC AAG GT
up (R)	AAG ACG GGG ACA GCA TTA GT
5' (F)	CGA GAA GAC TCC CGT GAA GA
5' (R)	AGG CGG CAA CAG CTT TAG TA
TES (F)	CCC ACC GCT CT CAG TAA AAG
TES (R)	ACT CCT CTC CCC GAC TTT GT
3' (F)	GCA GTG AGG GAC TTC AGA GG
3' (R)	GCA GTA GTC TCC CCA AGC AC
JUNB	
up (F)	CAC AAA CCC CTG TCA CCT TG
up (R)	TCT GGT CTG TCA CTG TGT CC
prom (F)	ATA AAG GCG TGT GGC TCA G
prom (R)	ATA GCT TTC CTG GCG TCG TT
GB (F)	TGC ACA AGA TGA ACC ACG TG
GB (R)	GCT GAG GTT GGT GTA AAC GG
TES (F)	TTA ACA GGG AGG GGA AGA GG
TES (R)	TTC CAC AGT ACG GTG CAG AG
3' (F)	AGG GAC CCA GGA GCT GAA G
3' (R)	GGC AGA ATC GGT CCT TGT AT
TFF1	
prom (F)	TAT GAA TCA CTT CTG CAG TGA G
prom (R)	GAG CGT TAG ATA ACA TTT GCC
RNU1	
N (F)	GCT GGC CTG TGT CAA TGA AA
N (R)	GCA GAT GTA ACC GCC TGA TG
A (F)	TGA GAA TCC CAG CTG TGT GT

A (R)	GCC TCA TAC GCC TCA CTC TT
B (F)	GGA GGC CTG GGG AAT AAG AA
B (R)	GAA ATG GAA GAG CTG AGC GG
RNU2	
N (F)	GGA GCG GAG CGT TCT CTG TCT CCC C
N (R)	AGA GTG TGA GCC CTC ATT CAC GCC C
P (F)	ATG AGA GTG GGA CGG TGA
P (R)	CAC TTG ATC TTA GCC AAA AGG
**mRNA**	
AQR (F)	ATG AAG ACA CCG AGG AAG CA
AQR (R)	CTG CCA CAT CTG TTT TGC CT
GAPDH (F)	ACA TCA AGA AGG TGG TGA AG
GAPDH (R)	GGG TCT TAC TCC TTG GAG GC
IFNGR1 (F)	GCC ACA GGT CCC TGT TTT TA
IFNGR1 (R)	TCC AAC CCT GGC TTT AAC TC
ISG20 (F)	TAG CCG CTC ATG TCC TCT TT
ISG20 (R)	TGA GGG AGA GAT CAC CGA TT
OAS1 (F)	CGC CTA GTC AAG CAC TGG TA
OAS1 (R)	CAG GAG CTC CAG GGC ATA
PTGS2 (F)	CCG GGT ACA ATC GCA CTT AT
PTGS2 (R)	GGC GCT CAG CCA TAC AG
RNASEH1 (F)	TCT TCC GAT TGT TTA GCT CCT TC
RNASEH1 (R)	TAA CTG GGT TCA AGG TTG GAA G
RNASEH2A (F)	AAT GGA GGA CAC GGA CTT TG
RNASEH2A (R)	GTG ACA GGG AGT TCA GGT TGT
RNASEH2B (F)	GAG GTC TTC TCC ATT TTG CCA C
RNASEH2B (R)	TGA TCA AGG GGC TGA AAC TTC C
RNASEH2C (F)	AAA GTG CGT GGG GCC TTA ACT
RNASEH2C (R)	GGT GCT GTG AAG AGC TCC TTT A
SELENBP1 (F)	CCA TCC AGC GCT TCT ACA AG
SELENBP1 (R)	CAG CCA GCC CTT CAC TTT C
STING (F)	CAT GGG CTG GCA TGG TCA TA
STING (R)	ATA TAC AGC CGC TGG CTC AC
TNF (F)	CCT CTC TCT AAT CAG CCC TCT G
TNF (R)	GAG GAC CTG GGA GTA GAT GAG
TUBG2 (F)	GAG AAA ATT CAT GAA GAC ATC
TUBG2 (R)	GCG ATG GAG TGA CAC AGC ACG

Supplementary References

1. Boguslawski, S.J. et al. Characterization of monoclonal antibody to DNA.RNA and its application to immunodetection of hybrids. *J Immunol Methods* **89**, 123-30 (1986).
2. Tellier, M. & Murphy, S. Incomplete removal of ribosomal RNA can affect chromatin RNA-seq data analysis. *Transcription*, 1-6 (2020).
3. Martin, M. Cutadapt removes adapter sequences from high-throughput sequencing reads. *EMBnet. journal* **17**, 10-12 (2011).
4. Dobin, A. et al. STAR: ultrafast universal RNA-seq aligner. *Bioinformatics* **29**, 15-21 (2013).
5. Li, H. et al. The Sequence Alignment/Map format and SAMtools. *Bioinformatics* **25**, 2078-9 (2009).
6. Ramirez, F. et al. deepTools2: a next generation web server for deep-sequencing data analysis. *Nucleic Acids Res* **44**, W160-5 (2016).
7. Zhang, Y. et al. Model-based analysis of ChIP-Seq (MACS). *Genome Biol* **9**, R137 (2008).
