# Supplementary material for: RNase H2, mutated in Aicardi‐Goutières syndrome, resolves co-transcriptional R-loops to prevent DNA breaks and inflammation
Source: Nat Commun. 2022 May 26;13:2961. doi: 10.1038/s41467-022-30604-0 (PMC9135716; doi:10.1038/s41467-022-30604-0)
Supplement: Supplementary file 3 — Reporting Summary [file 41467_2022_30604_MOESM3_ESM.pdf]

nature portfolio

Corresponding author(s): GROMAK N

Last updated by author(s): Apr 27, 2022

Reporting Summary

Nature Portfolio wishes to improve the reproducibility of the work that we publish. This form provides structure for consistency and transparency in reporting. For further information on Nature Portfolio policies, see our Editorial Policies and the Editorial Policy Checklist.

Statistics

For all statistical analyses, confirm that the following items are present in the figure legend, table legend, main text, or Methods section.

n/a	Confirmed	
☐	☒	The exact sample size (*n*) for each experimental group/condition, given as a discrete number and unit of measurement
☐	☒	A statement on whether measurements were taken from distinct samples or whether the same sample was measured repeatedly
☐	☒	The statistical test(s) used AND whether they are one- or two-sided<br>*Only common tests should be described solely by name; describe more complex techniques in the Methods section.*
☒	☐	A description of all covariates tested
☐	☒	A description of any assumptions or corrections, such as tests of normality and adjustment for multiple comparisons
☐	☒	A full description of the statistical parameters including central tendency (e.g. means) or other basic estimates (e.g. regression coefficient) AND variation (e.g. standard deviation) or associated estimates of uncertainty (e.g. confidence intervals)
☐	☒	For null hypothesis testing, the test statistic (e.g. *F*, *t*, *r*) with confidence intervals, effect sizes, degrees of freedom and *P* value noted<br>*Give P values as exact values whenever suitable.*
☒	☐	For Bayesian analysis, information on the choice of priors and Markov chain Monte Carlo settings
☒	☐	For hierarchical and complex designs, identification of the appropriate level for tests and full reporting of outcomes
☒	☐	Estimates of effect sizes (e.g. Cohen's *d*, Pearson's *r*), indicating how they were calculated

Our web collection on statistics for biologists contains articles on many of the points above.

Software and code

Policy information about availability of computer code

Data collection	Microscopy images were acquired with a LSM 880 microscope (ZEISS) with the objective Plan-Apochromat 63x/1.4 Oil DIC M27.<br>For authomated analysis in 96-well plates, it was used an Operetta High-Content Imaging System (PerkinElmer), 20X objective, with Harmony software (version 4.9).<br>Comet images were acquired with an AxioObserver Z1 fluorescence microscope (ZEISS) with the objective EC Plan-Neofluar 10X / 0.3 Ph1.<br>Real-time quantitative PCRs (qPCRs) were performed on a Rotor Gene Q Thermocycler (QIAGEN) with the softaware v2.3.1 or with a CFX96TM Real-time System (Bio-Rad) using the Bio-Rad CFX Manager 3.1.<br>Some of the western blotting were acquired with a ChemiDoc MP Imaging System (Bio-Rad) with the Image Lab software 4.1<br>STAR version 2.6.1d was used to map the trimmed reads to the human genome or the rRNA repeat sequence.<br>Manipulation of the bam files was done with SAMtools version 1.3.1.<br>PCR deduplication was performed with Picard MarkDuplicates tool.<br>ChIP-seq and DIP-seq peak calling were performed with MACS2 version 2.1.1.20160309.<br>The creation of bigwig files and generation of metagene profiles were performed with deepTools version 2.5.0.1.
Data analysis	Western blots bands were quantified using Image Studio Lite software v5.2 (Li-COR Biosciences) or Image Lab Software 6.1 (Bio-Rad).<br>Quantification of immunofluorescence was done by using ImageJ (version 1.52p) or Columbus software (version 2.8.2).<br>Comet analysis was performed on ImageJ (version 1.52p) with the plugin OpenComet (http://www.cometbio.org) or using a macro provided by Robert Bagnell (https://www.med.unc.edu/microscopy/resources/imagej-plugins-and-macros/comet-assay) as in (Mamouni et al., Mol Cell Biol, 2014 PMID:24912678 and Cristini et al., Nucleic Acids Res, 2016 PMID:26578593).<br>Display of plots and statistical analyses were carried out using the Prism software (GraphPad) v8.3.1 or v9.1.

For manuscripts utilizing custom algorithms or software that are central to the research but not yet described in published literature, software must be made available to editors and reviewers. We strongly encourage code deposition in a community repository (e.g. GitHub). See the Nature Portfolio guidelines for submitting code & software for further information.

Data

Policy information about availability of data

All manuscripts must include a data availability statement. This statement should provide the following information, where applicable:

- Accession codes, unique identifiers, or web links for publicly available datasets
- A description of any restrictions on data availability
- For clinical datasets or third party data, please ensure that the statement adheres to our policy

ChIP-seq, DRIP-seq and chrRNA-seq generated in this study have been deposited in the NCBI GEO database under accession code GSE146970. Protein-coding genes annotation was taken from Gencode V31 and eRNA from FANTOM5, both based on hg38 version of the human genome. Human ribosomal DNA complete repeating unit (GenBank: U13369.1) was used for rRNA read mapping. Bed files for called peaks are available in the GEO submission GSE146970.

Field-specific reporting

Please select the one below that is the best fit for your research. If you are not sure, read the appropriate sections before making your selection.

☒ Life sciences ☐ Behavioural & social sciences ☐ Ecological, evolutionary & environmental sciences

For a reference copy of the document with all sections, see nature.com/documents/nr-reporting-summary-flat.pdf

Life sciences study design

All studies must disclose on these points even when the disclosure is negative.

Sample size	No calculation or statistics were used to determine sample size. Sample size was determined according to accepted standards in the field. For immunofluorescence experiments, at least 50-100 cells per individual sample in manual analysis and 10,000 for automated analysis were counted for statistical analysis and at least 400 nuclei were analysed for COMET experiments. Statistical analysis was used to determine statistical significance of obtained results (as indicated in figure legends). p-values are reported in figures and/or figure legends.
Data exclusions	No samples were excluded.
Replication	Genomic experiments for RNase H2A ChIP-seq, DRIP-seq (with the exception of RNase H control that was performed once) and chrRNA-Seq were performed twice (n=2 biologically independent experiments). All attempts of replication were successful. ChIP- and DRIP-qPCR validation confirmed corresponding sequencing results. Pol II ChIP-seq was performed once and compared with previous RNA Pol II ChIP-seq in HeLa cells. Biologically independent experiments were performed as stated in the corresponding figure legends. The main observations were reproduced in two human cell lines (HeLa and HEK293T). As indicated in the figure legends, minimal size for ChIP-qPCR, DRIP-qPCR and RT-qPCR was n=2 biologically independent experiments.
Randomization	Randomization was not feasible for this type of molecular biology, biochemistry and genomics experiments. All samples in the same experiments were treated in the same manner.
Blinding	In microscopy analysis, the same settings between samples were used for data collection and analysis. With automated immunoflorescence analysis, data collection and analysis were performed automatically with no need for blinding. In the other experiments, blinding was not possible as data collection and analysis were performed by the same person.

Behavioural & social sciences study design

All studies must disclose on these points even when the disclosure is negative.

Study description	*Briefly describe the study type including whether data are quantitative, qualitative, or mixed-methods (e.g. qualitative cross-sectional, quantitative experimental, mixed-methods case study).*
Research sample	*State the research sample (e.g. Harvard university undergraduates, villagers in rural India) and provide relevant demographic information (e.g. age, sex) and indicate whether the sample is representative. Provide a rationale for the study sample chosen. For studies involving existing datasets, please describe the dataset and source.*
Sampling strategy	*Describe the sampling procedure (e.g. random, snowball, stratified, convenience). Describe the statistical methods that were used to predetermine sample size OR if no sample-size calculation was performed, describe how sample sizes were chosen and provide a rationale for why these sample sizes are sufficient. For qualitative data, please indicate whether data saturation was considered, and what criteria were used to decide that no further sampling was needed.*
Data collection	*Provide details about the data collection procedure, including the instruments or devices used to record the data (e.g. pen and paper, computer, eye tracker, video or audio equipment) whether anyone was present besides the participant(s) and the researcher, and whether the researcher was blind to experimental condition and/or the study hypothesis during data collection.*
Timing	*Indicate the start and stop dates of data collection. If there is a gap between collection periods, state the dates for each sample*

Timing	*cohort.*
Data exclusions	*If no data were excluded from the analyses, state so OR if data were excluded, provide the exact number of exclusions and the rationale behind them, indicating whether exclusion criteria were pre-established.*
Non-participation	*State how many participants dropped out/declined participation and the reason(s) given OR provide response rate OR state that no participants dropped out/declined participation.*
Randomization	*If participants were not allocated into experimental groups, state so OR describe how participants were allocated to groups, and if allocation was not random, describe how covariates were controlled.*

Ecological, evolutionary & environmental sciences study design

All studies must disclose on these points even when the disclosure is negative.

Study description	*Briefly describe the study. For quantitative data include treatment factors and interactions, design structure (e.g. factorial, nested, hierarchical), nature and number of experimental units and replicates.*
Research sample	*Describe the research sample (e.g. a group of tagged Passer domesticus, all Stenocereus thurberi within Organ Pipe Cactus National Monument), and provide a rationale for the sample choice. When relevant, describe the organism taxa, source, sex, age range and any manipulations. State what population the sample is meant to represent when applicable. For studies involving existing datasets, describe the data and its source.*
Sampling strategy	*Note the sampling procedure. Describe the statistical methods that were used to predetermine sample size OR if no sample-size calculation was performed, describe how sample sizes were chosen and provide a rationale for why these sample sizes are sufficient.*
Data collection	*Describe the data collection procedure, including who recorded the data and how.*
Timing and spatial scale	*Indicate the start and stop dates of data collection, noting the frequency and periodicity of sampling and providing a rationale for these choices. If there is a gap between collection periods, state the dates for each sample cohort. Specify the spatial scale from which the data are taken*
Data exclusions	*If no data were excluded from the analyses, state so OR if data were excluded, describe the exclusions and the rationale behind them, indicating whether exclusion criteria were pre-established.*
Reproducibility	*Describe the measures taken to verify the reproducibility of experimental findings. For each experiment, note whether any attempts to repeat the experiment failed OR state that all attempts to repeat the experiment were successful.*
Randomization	*Describe how samples/organisms/participants were allocated into groups. If allocation was not random, describe how covariates were controlled. If this is not relevant to your study, explain why.*
Blinding	*Describe the extent of blinding used during data acquisition and analysis. If blinding was not possible, describe why OR explain why blinding was not relevant to your study.*

Did the study involve field work? ☐ Yes ☐ No

Field work, collection and transport

Field conditions	*Describe the study conditions for field work, providing relevant parameters (e.g. temperature, rainfall).*
Location	*State the location of the sampling or experiment, providing relevant parameters (e.g. latitude and longitude, elevation, water depth).*
Access & import/export	*Describe the efforts you have made to access habitats and to collect and import/export your samples in a responsible manner and in compliance with local, national and international laws, noting any permits that were obtained (give the name of the issuing authority, the date of issue, and any identifying information).*
Disturbance	*Describe any disturbance caused by the study and how it was minimized.*

Reporting for specific materials, systems and methods

We require information from authors about some types of materials, experimental systems and methods used in many studies. Here, indicate whether each material, system or method listed is relevant to your study. If you are not sure if a list item applies to your research, read the appropriate section before selecting a response.

Materials & experimental systems

n/a	Involved in the study
☐	☒ Antibodies
☐	☒ Eukaryotic cell lines
☒	☐ Palaeontology and archaeology
☒	☐ Animals and other organisms
☒	☐ Human research participants
☒	☐ Clinical data
☒	☐ Dual use research of concern

Methods

n/a	Involved in the study
☐	☒ ChIP-seq
☒	☐ Flow cytometry
☒	☐ MRI-based neuroimaging

Antibodies

Antibodies used

Donkey anti-Rabbit IgG (H+L) Highly Cross-Adsorbed Secondary Antibody, Alexa Fluor 594 (Thermo Fisher Scientific Cat# A-21207, RRID:AB_141637). IF 1:500.
Goat anti-Rabbit IgG (H+L) Highly Cross-Adsorbed Secondary Antibody, Alexa Fluor 647 (Thermo Fisher Scientific Cat# A-21245, RRID:AB_2535813). IF 1:1000.
Goat anti-Mouse IgG (H+L) Cross-Adsorbed Secondary Antibody, Alexa Fluor 488 (Thermo Fisher Scientific Cat# A-11001, RRID:AB_2534069). IF 1:500.
AQR (Proteintech Cat# 24342-1-AP, RRID:AB_2879501). WB 1:500.
BrdU clone B44 (BD Biosciences Cat# 347580, RRID:AB_400326). IF 1:50.
FLAG clone M2 (Sigma-Aldrich Cat# F3165, RRID:AB_259529). WB 1:2000.
H3 (Abcam Cat# ab1791, RRID:AB_302613). WB 1:4000.
H3 clone D1H2 (Cell Signaling Technology Cat# 4499, RRID:AB_10544537). WB 1:1000.
IgG Isotype Control (Thermo Fisher Scientific Cat# 02-6102, RRID:AB_2532938). Co-IP 2 µg.
Lamin B1 (Abcam Cat# ab16048, RRID:AB_443298). WB 1:2000.
Nucleolin (Abcam Cat# ab22758, RRID:AB_776878). IF and WB 1:1000.
Pol I RPA194 clone C-1 (Santa Cruz Biotechnology Cat# sc-48385, RRID:AB_675814). WB 1:500.
Pol II (Novus, NBP2-32080). ChIP 1µg.
Pol II clone 8WG16 (Abcam Cat# ab817, RRID:AB_306327). IF 1:500 and WB 1:1000.
Pol II clone CTD4H8 (Santa Cruz Biotechnology Cat# sc-47701, RRID:AB_677353). WB 1:1000.
Pol II-S5P (Abcam Cat# ab5131, RRID:AB_449369). WB 1:2000.
Pol II-S2P (Abcam Cat# ab5095, RRID:AB_304749). WB 1:2000.
RNA/DNA hybrids clone S9.6 (Gromak Lab, University of Oxford Cat# Gromak_1, RRID:AB_2810829). DRIP 15 µl/sample.
RNase H1 (Proteintech Cat# 15606-1-AP, RRID:AB_2238624). WB 1:1000.
RNase H2A (Proteintech Cat# 16132-1-AP, RRID:AB_2269729). WB 1:1000, ChIP 2.5 µg, ChIP-seq 4 µg and co-IP 2 µg.
RNase H2C (Proteintech Cat# 16518-1-AP, RRID:AB_2181648). WB 1:500 and ChIP 2.5 µg and co-IP 2 µg.
Tubulin (Abcam, Cat#ab4074, RRID:AB_2288001). WB 1:5000.
XPF (Abcam Cat# ab76948, RRID:AB_1524575). WB 1:500.
XPG (Proteintech Cat# 11331-1-AP, RRID:AB_2098155). WB 1:500.

Validation

Rabbit polyclonal AQR antibody was validated by Proteintech using WB (1:1500 dilution) in HEK293 and Jurkat cell extracts. Specific WB band was detected after IP of AQR from HEK293 cell lysates compared to no band from IgG control IP. https://www.ptglab.com/products/AQR-Antibody-24342-1-AP.htm. We further validated the specificity of AQR antibody by WB of AQR upon siRNA-mediated knock-down in HeLa cells (Fig S7d).

Mouse monoclonal BrdU, clone B44, was described by BD Bioscience in mouse and human cells for flow cytometry and IF applications. https://www.bdbiosciences.com/content/bdb/paths/generate-tds-document.us.347580.pdf. This antibody has been previously used by the authors in Cristini et al, NAR, 2016 PMID: 26578593. We further validated the specificity of anti-BrdU antibody for IF by using aphidicolin, which inhibits DNA replication and thus, incorporation of BrdU in the newly synthesized DNA (Fig S2c-d).

Mouse monoclonal FLAG antibody, clone M2, is recommended for WB application by the Sigma-Aldrich. This antibody has been previously used by the authors for WB in Béry et al, Nat Commun, 2020 PMID: 32591521. We further validated the specificity by inducing the expression of RNase H1-FLAG in comparison to non-induced control (Fig 4g).

Rabbit polyclonal H3 antibody was validated by Abcam using WB (1:1000 dilution) in A431, Jurkat, HEK293 and HeLa whole cell extracts. The specific WB band in HEK293 and Jurkat can be prevented by incubation with human histone H3 peptide. Specific WB band is detected after IP of H3 from HeLa whole cell lysates compared to no band from "no antibody" control IP. It was also validated for usage as a nuclear fraction control in mouse cells by Abcam. https://www.abcam.com/histone-h3-antibody-nuclear-marker-and-chip-grade-ab1791.html. This antibody has been previously used as a nuclear marker control by the authors in Cristini et al, NAR, 2016 PMID: 26578593.

Rabbit monoclonal H3 antibody was validated by Cell Signalling Technology for WB (1:2000 dilution) in HeLa cell extracts. This antibody does not cross-react with other core histones. https://www.cellsignal.com/products/primary-antibodies/histone-h3-d1h2-xp-rabbit-mab/4499.

Rabbit IgG Isotype control was used in literature as IP control (e.g. Ge Zhy, Mol Cell, 2019 PMID: 30846317). We further showed the absence of protein binding tested by WB following co-IP with this antibody (Fig 3a, and S3a-c).

Rabbit polyclonal Lamin B1 antibody was validated by Abcam using WB (0.1-1 µg/ml dilution) in HeLa and A431 whole cell extracts.

No band was detected upon LMNB1 (lamin B1) knock-out in HAP1 cells. https://www.abcam.com/lamin-b1-antibody-nuclear-envelope-marker-ab16048.html.

Rabbit polyclonal Nucleolin antibody was validated by Abcam using WB (1 µg/ml dilution) in Jurkat and HAP1 whole cell extracts. Specific WB band was detected following IP of nucleolin from Jurkat whole cell lysates compared to no band from “no antibody” control IP. IF application of this antibody at 1 µg/ml dilution has been validated by Abcam in HeLa cells treated with the transcription inhibitor Triptolide showing changes in the nuclear localization of the nucleolin. https://www.abcam.com/nucleolin-antibody-ab22758.html.

Monoclonal Pol I RPA194 clone C-1 was validated by Santa Cruz using WB in WI-38 and ES-2 whole cell extracts. Specificity was confirmed by the WB band corresponding to the overexpressed truncated human RPA194 form in HEK293T cells. https://www.scbt.com/it/p/rpa194-antibody-c-1.

Rabbit polyclonal Pol II NBP2-32080 antibody was validated for ChIP application by Novus Biologicals in HeLa cells by comparing the ChIP-qPCR recovery at JUNB gene with this antibody to other commercial Pol II antibodies and to the IgG negative control. https://www.novusbio.com/products/rna-polymerase-ii-polr2a-antibody_nbp2-32080. This antibody has been previously used by the authors in Tellier et al, NAR, 2018 PMID: 30329085.

Monoclonal mouse Pol II clone 8WG16 was validated by Abcam using WB (0.25 µg/ml dilution) in HeLa and THP-1 whole cell extracts. IF application of this antibody at 2 µg/ml dilution has been validated by Abcam in HeLa cells. https://www.abcam.com/rna-polymerase-ii-ctd-repeat-ysptsps-antibody-8wg16-chip-grade-ab817.html.

Monoclonal Pol II clone CTD4H8 was validated by Santa Cruz using WB in A431, A673, U2OS, HeLa and Jurkat whole cell extracts. Specific WB band following IP of Pol II from A431 cell lysates was detected. https://www.scbt.com/it/p/pol-ii-antibody-ctd4h8.
Rabbit polyclonal Pol II-S5P was validated by Abcam using WB (1 µg/ml dilution) in HeLa nuclear cell extracts. The specific WB band in HeLa cells can be prevented by incubation with human RNA polymerase II CTD repeat YSPTSPS (phospho S5). https://www.abcam.com/rna-polymerase-ii-ctd-repeat-ysptsps-phospho-s5-antibody-ab5131.html.

Rabbit polyclonal Pol II-S2P was validated by Abcam using WB (1 µg/ml dilution) in HeLa whole cell extracts. The specific WB band in HeLa cells can be prevented by incubation with S. cerevisiae RNA polymerase II CTD repeat YSPTSPS (phospho S2) peptide. https://www.abcam.com/rna-polymerase-ii-ctd-repeat-ysptsps-phospho-s2-antibody-ab5095.html.

The mouse monoclonal RNA/DNA hybrid clone S9.6 antibody used in this study was previously validated for DRIP application by showing that RNase H digestion significantly removed S9.6 signal, indicating its specificity for RNA/DNA hybrids (Groh et al, PLoS Genet, 2014 PMID: 24787137; Cristini et al, Cell Rep, 2018 PMID:29742442; Cristini et al, Cell Rep, 2019 PMID: 31533039). We further validated the specificity of this antibody in our DRIP-seq sequencing by using RNase H digestion in vitro control (Fig. 4d) and in DRIP-qPCR by using RNase H1 overexpression in HEK293T cells (Fig 4h).

Rabbit polyclonal RNase H1 antibody was validated by Proteintech using WB (1:500) in HeLa cell extracts. Specific WB band following IP of RNase H1 from HeLa cell lysates was detected compared to no band from IgG control IP. https://www.ptglab.com/products/RNASEH1-Antibody-15606-1-AP.htm. We further validated the specificity of RNase H1 antibody by WB upon RNase H1 overexpression in HEK293T cells (Fig 4g).

Rabbit polyclonal RNase H2A antibody was validated by Proteintech using WB (1:500 dilution) in HeLa and human liver tissue extracts. Specific WB band was detected following IP of RNase H2A from human liver tissue lysates compared to no band from IgG control IP. https://www.ptglab.com/products/RNASEH2A-Antibody-16132-1-AP.htm. We validated the IP application in HeLa and WI-38 cells (Fig 3a and S3b). We further validated the specificity of RNase H2A antibody for WB and ChIP applications in HeLa cells, showing the decreased antibody signal upon siRNA-mediated knock-down in HeLa cells (Fig 1a and Fig 1h, j, l).

Rabbit polyclonal RNase H2C antibody was validated by Proteintech using WB (1:300 dilution) in HeLa and HEK293 cell extracts. Specific WB band was detected following IP of RNase H2C from HEK293 lysates compared to no band from IgG control IP. https://www.ptglab.com/products/RNASEH2C-Antibody-16518-1-AP.htm. We validated the IP application in HeLa cells (Fig S3a). We further validated the specificity of RNase H2C antibody for WB, showing the decreased antibody signal upon siRNA-mediated knock-down in HeLa and HEK293T cells (Fig 1a and Fig 4g). confirm

Rabbit polyclonal alpha Tubulin was validated by Abcam using WB (1 µg/ml dilution) in HeLa and HEK293 whole cell extracts. The specific WB band in HeLa and HEK293 can be prevented by incubation with human alpha-tubulin peptide. https://www.abcam.com/alpha-tubulin-antibody-loading-control-ab4074.html.

Rabbit polyclonal XPF antibody was validated by Abcam using WB (1:2000 dilution) in HeLa and HEK293 cell extracts. No band at 105 kDa was detected upon ERCC4 (XPF) knock-out in HeLa cells. Specific WB band was detected following IP of XPF from HeLa cell lysates. https://www.abcam.com/xpf-antibody-ab76948.html. We further validated the specificity of XPF antibody by WB of XPF upon siRNA-mediated knock-down in HeLa cells (Fig S8e).

Rabbit polyclonal XPG antibody was validated by Proteintech using WB (1:1500 dilution) in HeLa, HepG2 and Daudi cell extracts. Specific WB band was detected following IP of XPG from HeLa cell lysates compared to no band from IgG control IP. https://www.ptglab.com/products/ERCC5-Antibody-11331-1-AP.htm We further validated the specificity of XPG antibody by WB of XPG upon siRNA-mediated knock-down in HeLa cells (Fig S8b).

Eukaryotic cell lines

Policy information about cell lines

Cell line source(s) | HeLa cells were obtained from Nicholas Proudfoot (University of Oxford, UK) and are originally from ATCC.

Cell line source(s)	HEK293T are from ATCC, Cat#CRL-3216 RRID:CVCL_0063<br>WI38 hTERT cells were obtained from Estelle Nicolas (LBCMCP, Toulouse, France) and Carl Mann (CEA, Gif-sur-Yvette, France) (Jeanblanc et al, Oncogene, 2012 PMID: 22020327).
Authentication	Only standard cell lines were employed and showed the expected morphology and growth features.
Mycoplasma contamination	All cell lines tested negative for mycoplasma.
Commonly misidentified lines<br>(See ICLAC register)	No commonly misidentified cell lines were used in this study.

Palaeontology and Archaeology

Specimen provenance	*Provide provenance information for specimens and describe permits that were obtained for the work (including the name of the issuing authority, the date of issue, and any identifying information). Permits should encompass collection and, where applicable, export.*
Specimen deposition	*Indicate where the specimens have been deposited to permit free access by other researchers.*
Dating methods	*If new dates are provided, describe how they were obtained (e.g. collection, storage, sample pretreatment and measurement), where they were obtained (i.e. lab name), the calibration program and the protocol for quality assurance OR state that no new dates are provided.*

☐ Tick this box to confirm that the raw and calibrated dates are available in the paper or in Supplementary Information.

Ethics oversight	*Identify the organization(s) that approved or provided guidance on the study protocol, OR state that no ethical approval or guidance was required and explain why not.*

Note that full information on the approval of the study protocol must also be provided in the manuscript.

Animals and other organisms

Policy information about studies involving animals; ARRIVE guidelines recommended for reporting animal research

Laboratory animals	*For laboratory animals, report species, strain, sex and age OR state that the study did not involve laboratory animals.*
Wild animals	*Provide details on animals observed in or captured in the field; report species, sex and age where possible. Describe how animals were caught and transported and what happened to captive animals after the study (if killed, explain why and describe method; if released, say where and when) OR state that the study did not involve wild animals.*
Field-collected samples	*For laboratory work with field-collected samples, describe all relevant parameters such as housing, maintenance, temperature, photoperiod and end-of-experiment protocol OR state that the study did not involve samples collected from the field.*
Ethics oversight	*Identify the organization(s) that approved or provided guidance on the study protocol, OR state that no ethical approval or guidance was required and explain why not.*

Note that full information on the approval of the study protocol must also be provided in the manuscript.

Human research participants

Policy information about studies involving human research participants

Population characteristics	*Describe the covariate-relevant population characteristics of the human research participants (e.g. age, gender, genotypic information, past and current diagnosis and treatment categories). If you filled out the behavioural & social sciences study design questions and have nothing to add here, write "See above."*
Recruitment	*Describe how participants were recruited. Outline any potential self-selection bias or other biases that may be present and how these are likely to impact results.*
Ethics oversight	*Identify the organization(s) that approved the study protocol.*

Note that full information on the approval of the study protocol must also be provided in the manuscript.

Clinical data

Policy information about clinical studies
All manuscripts should comply with the ICMJE guidelines for publication of clinical research and a completed CONSORT checklist must be included with all submissions.

Clinical trial registration	*Provide the trial registration number from ClinicalTrials.gov or an equivalent agency.*
Study protocol	*Note where the full trial protocol can be accessed OR if not available, explain why.*
Data collection	*Describe the settings and locales of data collection, noting the time periods of recruitment and data collection.*

Outcomes

Describe how you pre-defined primary and secondary outcome measures and how you assessed these measures.

Dual use research of concern

Policy information about dual use research of concern

Hazards

Could the accidental, deliberate or reckless misuse of agents or technologies generated in the work, or the application of information presented in the manuscript, pose a threat to:

No	Yes	
☐	☐	Public health
☐	☐	National security
☐	☐	Crops and/or livestock
☐	☐	Ecosystems
☐	☐	Any other significant area

Experiments of concern

Does the work involve any of these experiments of concern:

No	Yes	
☐	☐	Demonstrate how to render a vaccine ineffective
☐	☐	Confer resistance to therapeutically useful antibiotics or antiviral agents
☐	☐	Enhance the virulence of a pathogen or render a nonpathogen virulent
☐	☐	Increase transmissibility of a pathogen
☐	☐	Alter the host range of a pathogen
☐	☐	Enable evasion of diagnostic/detection modalities
☐	☐	Enable the weaponization of a biological agent or toxin
☐	☐	Any other potentially harmful combination of experiments and agents

ChIP-seq

Data deposition

☒ Confirm that both raw and final processed data have been deposited in a public database such as GEO.

☒ Confirm that you have deposited or provided access to graph files (e.g. BED files) for the called peaks.

Data access links<br>*May remain private before publication.*	The GEO accession number for the chrRNA-seq, ChIP-seq and DRIP-seq reported in this paper is GSE146970 and is publicly accessible.
Files in database submission	ChrSeq_siH2A_R1, ChrSeq_siH2A_R2, ChrSeq_siLuc_R1, ChrSeq_siLuc_R2, DRIP_siH2A_R1, DRIP_siH2A_R2, DRIP_siH2A_RNaseH, DRIP_siLuc_R1, DRIP_siLuc_R2, DRIP_siLuc_RNaseH, Input_PolII, Input_RNaseH2A_R1, Input_RNaseH2A_R2, Input_siH2A_DRIP_R1, Input_siH2A_DRIP_R2, Input_siLuc_DRIP_R1, Input_siLuc_DRIP_R2, PolII, RNaseH2A_R1, RNaseH2A_R2. Raw data and processed files submitted for each sample.
Genome browser session<br>(e.g. UCSC)	https://genome.ucsc.edu/s/TMichael2/RNaseH2A_Data_NatureCommunications_hg38

Methodology

Replicates	Two biologically independent experiments were performed for the RNaseH2A ChIP-seq, DRIP-seq (except for the RNaseH treated samples, performed once) and Chromatin RNA-seq. Total RNA polymerase II ChIP-seq was performed only once but the quality of the data was confirmed based on other RNA polymerase II ChIP-seq experiments we obtained previously in HeLa cells.
Sequencing depth	ChIP-seq and DRIP-seq were performed with 75 bp paired-end reads. Chromatin RNA-seq were performed with 150 bp paired-end reads. Each chromatin RNA-seq experiment has more than 140 million properly mapped and paired-end reads. Each ChIP-seq experiment has more than 28 million properly mapped and paired-end reads. Each DRIP-seq experiment has more than 42 million properly mapped and paired-end reads.
Antibodies	RNase H2A (Proteintech Cat# 16132-1-AP, RRID:AB_2269729; Lot#00007351) for RNase H2A ChIP-seq<br>Pol II (Novus, NBP2-32080) for RNA polymerase II ChIP-seq<br>RNA/DNA hybrids (S9.6, Natalia Gromak Lab, University of Oxford Cat# Gromak_1, RRID:AB_2810829) for DRIP-seq

Peak calling parameters	Peak calling was performed with MACS version 2.1.1.20160309 with the following options: callpeak -t Treatment.bam (IP) -c Control.bam (Input) -f BAMPE -g 2.9e9 -B -q 0.01 -n Name_Output_Folder --call-summits
Data quality	Significant peak summits: fold enrichment > 5 and q-value < 0.01<br>Number of peak summits with for the DRIP-seq siLuc: 12,833<br>Number of peak summits with for the DRIP-seq siRNaseH2A: 8,539<br>Number of peak summits with for the ChIP-seq RNaseH2A: 5,135
Software	Adapters trimming was performed with Cutadapt v1.13. Mapping to the human genome was done with STAR v2.6.1d. SAMtools v1.3.1 was used to extract the properly paired and mapped reads and to index the bam files. Picardtools was used to remove the PCR duplicates. Bigwig files were generated with Deeptools2 v2.5.0.1.

Flow Cytometry

Plots

Confirm that:

- [ ] The axis labels state the marker and fluorochrome used (e.g. CD4-FITC).
- [ ] The axis scales are clearly visible. Include numbers along axes only for bottom left plot of group (a 'group' is an analysis of identical markers).
- [ ] All plots are contour plots with outliers or pseudocolor plots.
- [ ] A numerical value for number of cells or percentage (with statistics) is provided.

Methodology

Sample preparation	*Describe the sample preparation, detailing the biological source of the cells and any tissue processing steps used.*
Instrument	*Identify the instrument used for data collection, specifying make and model number.*
Software	*Describe the software used to collect and analyze the flow cytometry data. For custom code that has been deposited into a community repository, provide accession details.*
Cell population abundance	*Describe the abundance of the relevant cell populations within post-sort fractions, providing details on the purity of the samples and how it was determined.*
Gating strategy	*Describe the gating strategy used for all relevant experiments, specifying the preliminary FSC/SSC gates of the starting cell population, indicating where boundaries between "positive" and "negative" staining cell populations are defined.*

- [ ] Tick this box to confirm that a figure exemplifying the gating strategy is provided in the Supplementary Information.

Magnetic resonance imaging

Experimental design

Design type	*Indicate task or resting state; event-related or block design.*
Design specifications	*Specify the number of blocks, trials or experimental units per session and/or subject, and specify the length of each trial or block (if trials are blocked) and interval between trials.*
Behavioral performance measures	*State number and/or type of variables recorded (e.g. correct button press, response time) and what statistics were used to establish that the subjects were performing the task as expected (e.g. mean, range, and/or standard deviation across subjects).*

Acquisition

Imaging type(s)	*Specify: functional, structural, diffusion, perfusion.*
Field strength	*Specify in Tesla*
Sequence & imaging parameters	*Specify the pulse sequence type (gradient echo, spin echo, etc.), imaging type (EPI, spiral, etc.), field of view, matrix size, slice thickness, orientation and TE/TR/flip angle.*
Area of acquisition	*State whether a whole brain scan was used OR define the area of acquisition, describing how the region was determined.*

Diffusion MRI ☐ Used ☐ Not used

Preprocessing

Preprocessing software	*Provide detail on software version and revision number and on specific parameters (model/functions, brain extraction, segmentation, smoothing kernel size, etc.).*
Normalization	*If data were normalized/standardized, describe the approach(es): specify linear or non-linear and define image types used for transformation OR indicate that data were not normalized and explain rationale for lack of normalization.*
Normalization template	*Describe the template used for normalization/transformation, specifying subject space or group standardized space (e.g. original Talairach, MNI305, ICBM152) OR indicate that the data were not normalized.*
Noise and artifact removal	*Describe your procedure(s) for artifact and structured noise removal, specifying motion parameters, tissue signals and physiological signals (heart rate, respiration).*
Volume censoring	*Define your software and/or method and criteria for volume censoring, and state the extent of such censoring.*

Statistical modeling & inference

Model type and settings	*Specify type (mass univariate, multivariate, RSA, predictive, etc.) and describe essential details of the model at the first and second levels (e.g. fixed, random or mixed effects; drift or auto-correlation).*
Effect(s) tested	*Define precise effect in terms of the task or stimulus conditions instead of psychological concepts and indicate whether ANOVA or factorial designs were used.*

Specify type of analysis: ☐ Whole brain ☐ ROI-based ☐ Both

Statistic type for inference (See Eklund et al. 2016)	*Specify voxel-wise or cluster-wise and report all relevant parameters for cluster-wise methods.*
Correction	*Describe the type of correction and how it is obtained for multiple comparisons (e.g. FWE, FDR, permutation or Monte Carlo).*

Models & analysis

n/a	Involved in the study
☐	☐ Functional and/or effective connectivity
☐	☐ Graph analysis
☐	☐ Multivariate modeling or predictive analysis

Functional and/or effective connectivity	*Report the measures of dependence used and the model details (e.g. Pearson correlation, partial correlation, mutual information).*
Graph analysis	*Report the dependent variable and connectivity measure, specifying weighted graph or binarized graph, subject- or group-level, and the global and/or node summaries used (e.g. clustering coefficient, efficiency, etc.).*
Multivariate modeling and predictive analysis	*Specify independent variables, features extraction and dimension reduction, model, training and evaluation metrics.*
